# Fused Filament Fabrication of PEEK: A Review of Process-Structure-Property Relationships

**DOI:** 10.3390/polym12081665

**Published:** 2020-07-27

**Authors:** Ali Reza Zanjanijam, Ian Major, John G. Lyons, Ugo Lafont, Declan M. Devine

**Affiliations:** 1 Materials Research Institute, Athlone Institute of Technology, N37 HD68 Athlone, Ireland; azanjanijam@ait.ie (A.R.Z.); imajor@ait.ie (I.M.); 2 Faculty of Engineering and Informatics, Athlone Institute of Technology, N37 HD68 Athlone, Ireland; slyons@ait.ie; 3 European Space Technology and Research Centre, European Space Agency, Keplerlaan 1, 2201 AZ Noordwijk, The Netherland; ugo.lafont@esa.int

**Keywords:** PEEK, additive manufacturing, 3D printing, fused filament fabrication (FFF), international space station (ISS)

## Abstract

Poly (ether ether ketone) (PEEK) is a high-performance engineering thermoplastic polymer with potential for use in a variety of metal replacement applications due to its high strength to weight ratio. This combination of properties makes it an ideal material for use in the production of bespoke replacement parts for out-of-earth manufacturing purposes, in particular on the International Space Station (ISS). Additive manufacturing (AM) may be employed for the production of these parts, as it has enabled new fabrication pathways for articles with complex design considerations. However, AM of PEEK via fused filament fabrication (FFF) encounters significant challenges, mostly stemming from the semi crystalline nature of PEEK and its associated high melting temperature. This makes PEEK highly susceptible to changes in processing conditions which leads to a large reported variation in the literature on the final performance of PEEK. This has limited the adaption of FFF printing of PEEK in space applications where quality assurance and reproducibility are paramount. In recent years, several research studies have examined the effect of printing parameters on the performance of the 3D-printed PEEK parts. The aim of the current review is to provide comprehensive information in relation to the process-structure-property relationships in FFF 3D-printing of PEEK to provide a clear baseline to the research community and assesses its potential for space applications, including out-of-earth manufacturing.

## 1. Introduction

Additive Manufacturing (AM) is a technology based on the principle of layer-by-layer manufacturing, which allows the production of complicated polymeric, metallic, and ceramic parts. Employing this revolutionary method decreases cycle time and cost of product development [1,2]. Fabrication of polymeric parts via AM is generally carried out using one of three different processes which are stereolithography (SLA) [3,4], selective laser sintering (SLS) [5,6,7], and fused filament fabrication (FFF) [8,9,10]. In SLA, a photopolymer resin is cured using an energy source, mostly UV laser, to form a single layer of the desired object. SLA was the first 3D printing machine developed and typically gives the best resolution [2]. However, the resin employed must be UV curable, which limits the materials that can be employed using this technique. SLS employs a laser to sinter a polymer powder as a feedstock material. It has been used as a technique to overcome drawbacks of other methods [11,12]. However, expensive instruments are required when using SLS, which is a big obstacle for making products [13]. FFF, also known as fused deposition modelling (FDM), is the most commonly used AM technology for fabrication of thermoplastic polymer parts based on computer-aided design (CAD). In FFF process, a polymer filament is continuously fed to a liquefier, it is melted and then extruded through a nozzle to construct the layer-by-layer structure on a heated bed. This three-dimensional (3D) printing technique offers low manufacturing cost, flexibility in structural design, and supervision-free operation [14,15,16,17]. The most common polymers that are printed via FFF are acrylonitrile-butadiene-styrene (ABS) [18,19,20], and poly(lactic acid) (PLA) [21,22,23]. However, other polymers such as poly(ether imide) (PEI) [24,25,26], polycarbonate (PC) [27,28,29], polystyrene (PS) [30,31], polyamide (PA) [32,33,34], polypropylene (PP) [35,36,37],poly(vinyl alcohol) (PVA) [38,39,40,41], polyethylene (PE) [42,43], polycaprolactone (PCL) [44,45], polyphenylene sulfide (PPS) [46], and polymer blends [47,48,49,50,51] have been also studied.

**Figure 1:**
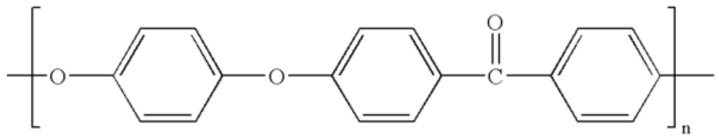
Chemical structure of poly (ether ether ketone) (PEEK) [60].

Poly(ether ether ketone) (PEEK) is an important member of poly (aryletherketone) (PAEK) family with many excellent characteristics. PEEK was first produced by Imperial Chemicals Industries in 1978, and since then, it has attracted a lot of attention in different industries [52]. Thanks to its linear aromatic structure (Figure 1), this high-performance thermoplastic polymer exhibits superior thermal resistance, mechanical performance [53], low linear expansion coefficient, and chemical stability (soluble only in sulphuric acid at 60 °C [54,55]. The physical and mechanical properties of PEEK are listed in Table 1. It has a melting temperature (T_m_) of 343 °C, glass transition temperature (T_g_) of 143 °C [56], and a service temperature up to 260 °C [57]. Having a high Young’s modulus and tensile strength along with a low specific gravity, allows PEEK to be used as a replacement of aluminium or steel in a wide range of applications, especially aerospace and automotive applications with an increasing interest in employing PEEK in the space industry [58,59]. Furthermore, since PEEK shows biocompatibility and radio-transparency, it is a promising material for biomedical applications, particularly as an appropriate alternative to precious metal implants [60,61,62,63,64,65,66,67,68]. It can also be sterilised repeatedly, which is ideal for surgical instruments and dental devices [64,69].

There are several methods used for processing PEEK-based products, including extrusion, injection moulding, compression moulding, machining, powder spraying, etc. [70,71,72,73]. The main shortcoming of these techniques is the lack of flexibility for making parts with complicated designs. It seems that additive manufacturing has the potential to develop PEEK parts without geometry limitations in a cost-effective way. Considering the melt processability of PEEK, both SLS and FFF can be employed for the production of 3D printed parts. SLS was the first AM process used for fabrication of PEEK [11]. However, it is more complicated and more expensive than FFF [74,75]. One of the limitations is in the recycling of non-fused PEEK powder, which increases processing expenditure [76]. Moreover, aiming to implement such process for out-of-earth manufacturing (in orbit or on planet), the effect of low gravity combined with health and safety aspect makes the use of powder less suitable. In this respect, FFF process that uses filament as the feedstock material has been successfully implemented in a low gravity environment on the International Space Station. Hence, there has been a growing interest in 3D printing of PEEK via FFF in recent years in situ production of parts on the ISS. However, a big challenge regarding 3D printed PEEK is its anisotropic mechanical properties, compared to PEEK parts produced using conventional processing methods such as injection moulding. As such, understanding the effects of different process parameters in additive manufacturing on the final properties of the printed parts, and thereby finding optimum process parameter levels are the key to fabricate PEEK end-parts with desirable, reproducible performance. The aim of this literature review is to address challenges and provide a comprehensive and practical guide to 3D printing of PEEK via FFF. This way, the focus will be on the process-structure-property relationships of the PEEK-based printed parts mostly based on tensile strength performances.

## 2. Challenges of 3D printing of PEEK

PEEK is a promising engineering polymer suitable for many high-performance applications. Recently, processing of PEEK through 3D printing has attracted a lot of attention. However, there are some barriers and difficulties in fabrication of ideal-performance parts. These barriers originate from both inherent issues of FFF such as thermal gradient, and specific physical properties of PEEK.

During layer-by-layer manufacturing, residual stress is accumulated, leading to warping and interlayer delamination [78,79]. These problems are more important for additive manufacturing of semi-crystalline polymers with a high melting temperature such as PEEK and can significantly affect dimensional accuracy and mechanical properties [80,81,82]. In fact, the degree of crystalline perfection of PEEK is considerably influenced by the rate of cooling and thermal gradient during crystallisation from melt [83]. Processing via FFF differs from injection moulding in many aspects, so the formation of defects in the parts and a subsequent loss in the final performance of printed polymers are expected. SEM micrograph of an injection moulded-PEEK is representative of a tough fracture surface, while 3D printed PEEK shows a smooth surface due to brittle fracture. Injection moulding provides an external pressure which decreases the internal defects leading to a better toughness. This is not the case in 3D printing, so inferior properties are observed for the printed samples [84].

**Figure 2:**
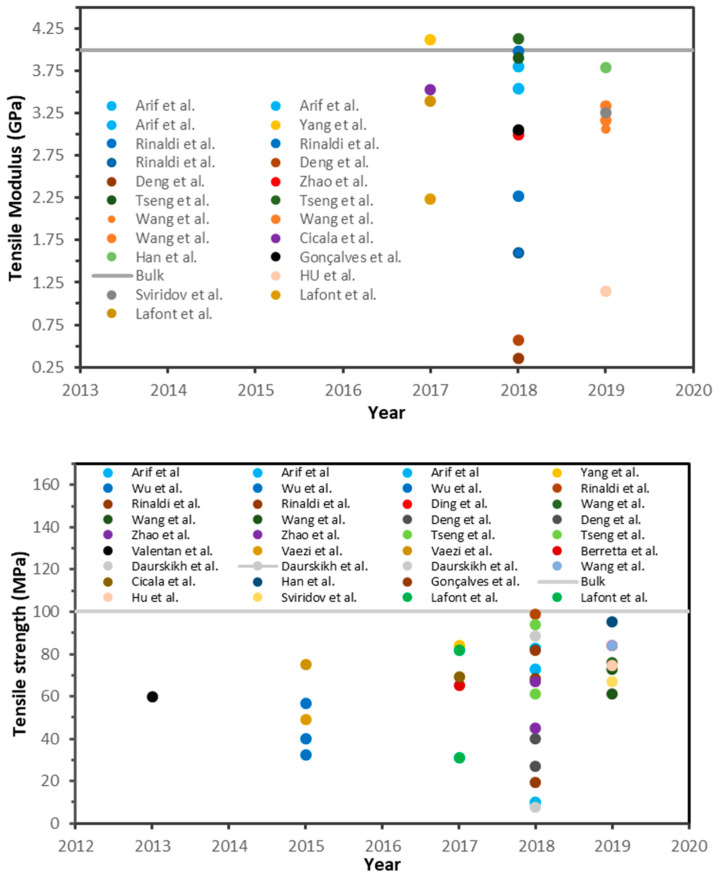

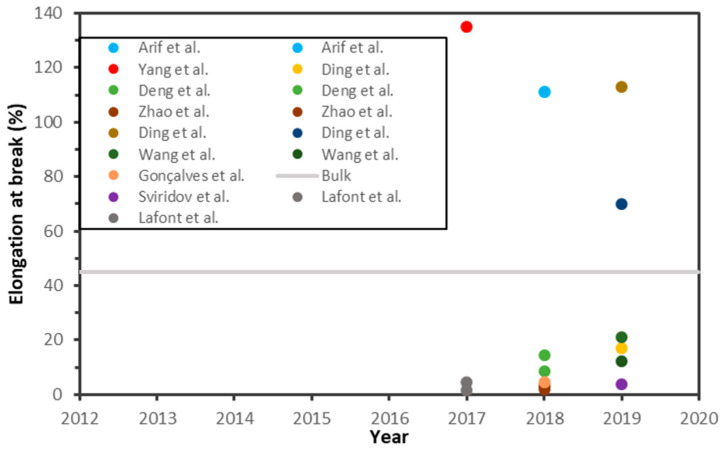
Tensile properties values for 3D-printed PEEK reported in the literature.

**Figure 3:**
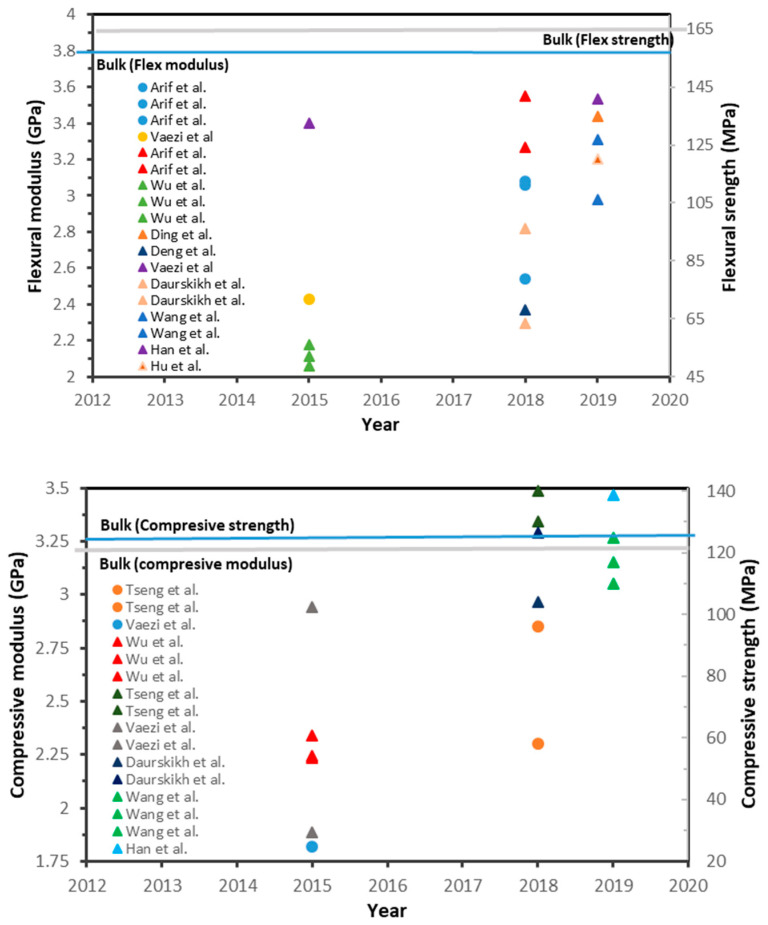
Flexural and compressive properties values for 3D-printed PEEK reported in the literature. Triangle and circle symbols are strength and modulus data, respectively.

To achieve the desired physical and mechanical properties for PEEK parts fabricated through FFF both polymer processing characteristics must be considered. For the former, knowledge of the rheological properties, crystallisation kinetics, etc. is required. As is knowledge of the additive manufacturing process in terms of ensuring the FFF printer is a compatible high-temperature printer and optimal printing parameters are known. To illustrate variances reported in the literature using non optimised conditions, Figure 2 shows the tensile properties of 3D-printed PEEK samples from various sources. A wide range of values can be seen for the mechanical properties in the reports. Most of the results are far from the properties of bulk material (processed via injection moulding). This large variation in the mechanical behaviour is due to different printing parameters such as printing orientation, nozzle temperature, environment temperature, bed temperature, printer type, PEEK type, etc. selected by the authors. Rinaldi et al. [85], Tseng et al. [86], Han et al. [87], and Yang et al. [88] reported values which approximately equalled those of injection moulded PEEK and in some cases exceed values for Young’s modulus and tensile strength. Flexural and compressive data from PEEK parts fabricated with FFF has also being reported albeit in fewer reports than for tensile properties. The compiled results for flexural and compression data of FFF 3D-printed PEEK are illustrated in Figure 3. According to the results, the values recorded for flexural modulus and flexural strength do not match those of injection moulded PEEK. In contrast, values for compressive strength of FFF3D-printed PEEK have being reported to exceed those of injection moulded parts. For example, Daurskikh et al. [89] and Wang et al. [90] obtained a compressive strength of 126.4 and 125 MPa, respectively. More interestingly, the compressive strength of PEEK samples in reports by Han et al. [87] and Tseng et al. [86] showed an 11% and 12% improvement in compressive strength relative to bulk PEEK, respectively.

A comparison of the additive manufacturing processing conditions from the literature reveals that optimal mechanical properties for FFF 3D-printed PEEK can be achieved by selecting a combination of appropriate printing parameters such as high printing temperature, bed temperature, and building direction. The parameters which have been reported in the literature for 3D printing of PEEK via FFF are listed in Table 2. For nozzle and bed, a wide range of temperatures have been reported. The values are in the range of 340–520 °C and 100–300 °C, respectively. The most common nozzle diameter is 0.4 mm, although nozzles with greater diameters have also been used in some cases. The authors also employed different commercial and modified/custom-build FFF printers. Table 3 summarises print conditions for commercial PEEK filaments recommended by the manufacturers. These parameters are merely for general use and may need to be altered depending on the FFF machine utilised. In fact, due to the large variety of printing machines, geometries and volumes of parts, etc., it may be necessary to employ different settings according to the filament/ FFF printer combination employed. As such, this review aims to give a comprehensive insight into the relationships between process, structure, and properties of PEEK parts produced using additive manufacturing. Additionally, the influence of printing path parameters and processing conditions on the performance of PEEK parts will be considered, in addition to the thermal stability of PEEK during 3D printing. The latter is critical when processing in enclosed environments as seen in the ISS as is the thermal stability of PEEK and its degradation in different conditions will also be considered.

## 3. Process-Structure-Property Relationships

### 3.1. Printing Path Parameters

**Figure 4:**
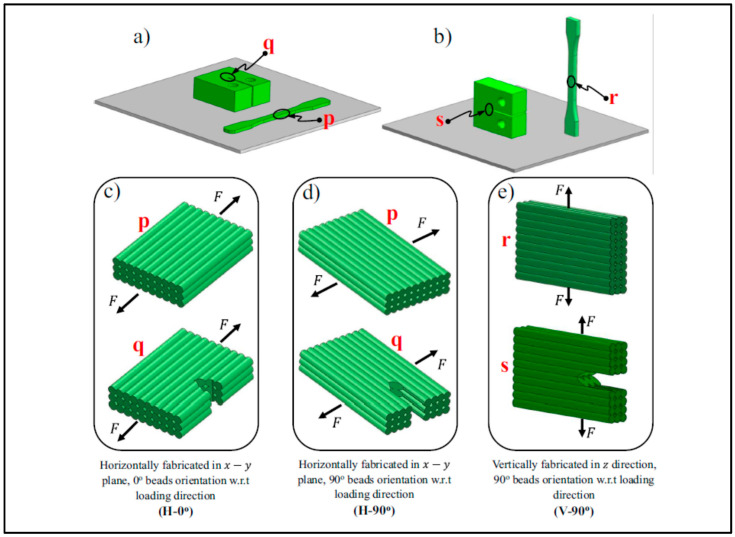
Configurations of samples fabricated (**a**) horizontally or (**b**) vertically. The deposition pattern is (**c**) 0° beads orientation or (**d**,**e**) 90° beads orientation [93].

**Figure 5:**
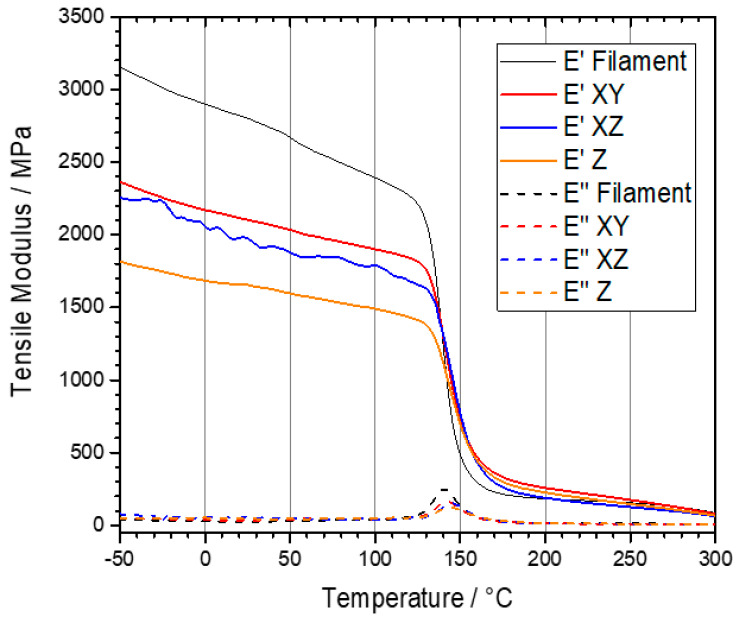
Storage and loss moduli of PEEK filament and 3D printed samples with a −45/+45 raster layer orientation [112].

3D printing could be performed in different building orientations and raster angles. These printing path parameters affect the microstructure and final properties of PEEK samples. Arif et al. [93] studied the effect of printing configurations and bead orientation on the mechanical behavior of PEEK processed by FFF. They evaluated the tensile and flexural properties of the samples printed horizontally and vertically with a raster angles of 0° (H-0°) and 90° (H-90°, V-90°), as shown in Figure 4. The H-0° and V-90° samples exhibited the best and worst mechanical properties, respectively. Similar results have been reported in other works focusing on the printing path parameters. When horizontal building orientation is used for printing, direction of applied tensile or flexural force is parallel to the orientation of the polymer filaments, and thereby the sample shows a ductile fracture. According to Table 4 which lists the results of mechanical properties of PEEK samples reported in the literature, near bulk mechanical properties can be achieved by printing in the horizontal direction. In contrast, the applied force during the test is normal to interface between layers for vertically printed samples leading to a brittle fracture. In fact, strength of interfacial bonding between beads is the key factor for these samples. Another interesting point is high standard deviation of the results for the parts printed at vertical direction as well as the difference between the reported values. The key role of the building orientation in determining final properties of PEEK can also be traced using dynamic mechanical analysis (DMA). Figure 5 shows the same trend for the samples printed in different orientations as discussed above. In enclosed environments with limited space as observed in the ISS the printing direction and raster angle, become paramount especially when printing large critical parts.

**Figure 6:**
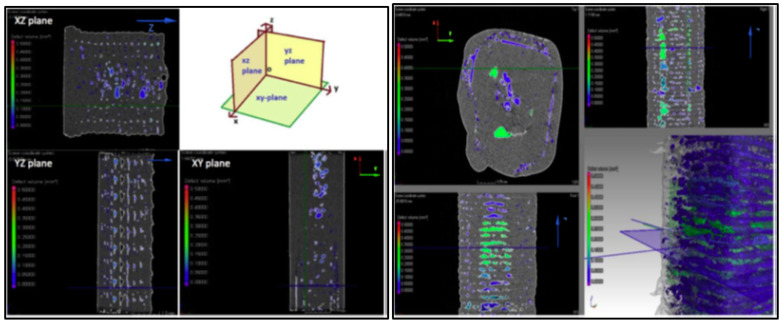
Computed Tomography scans of gauge length of PEEK-XY-100 (**left**) and PEEK-Z-100 (**right**) samples before tensile test [85].

**Figure 7:**
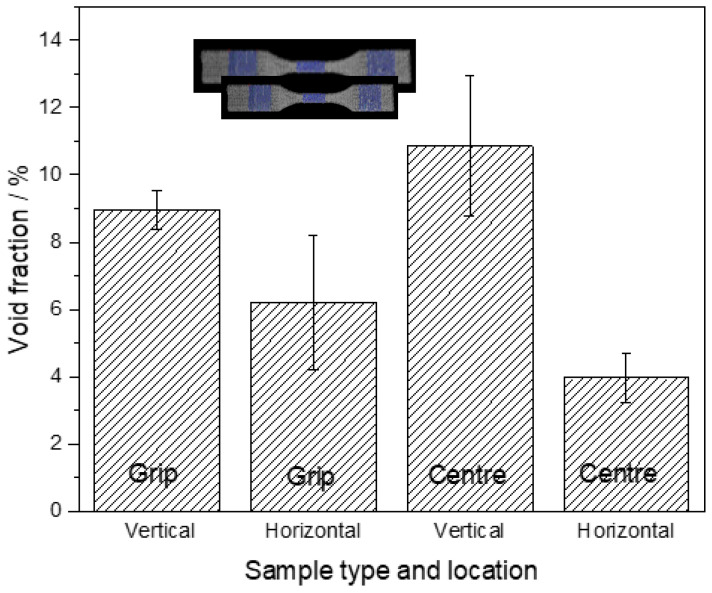
Void fraction calculated from CT Scan as a function of the printing orientation and scan location [110].

CT scans of PEEK based tensile test specimen before the test (Figure 6) reveals that of the void fraction in the samples printed horizontally and vertically is nearly same (6.7% and 7.6%, respectively). However, the size of the voids in the Z-printed samples is much larger than XY-samples. Additionally, the orientation of the voids with respect to the force direction can have a devastating effect on the properties of the printed part [85]. Another example by Lafont et al. confirms that vertical printed sample exhibit more voids than horizontal printed samples. Moreover, it is also interesting that the void fraction can differ depending on the investigated area (Figure 7). For example, in ASTM Type V specimen, CT scan results suggest that the narrow central part of the sample contains more voids than the grip area [106].

Results indicate that a raster angle of 0° (aligned in the direction of the tensile strength) can lead to better mechanical behaviour compared to 90° (Table 4). Although shorter bead deposition path, better polymer diffusion, and thereby stronger interfacial bonding between beads are conceivable for raster angle of 90°, however, the strength of the filaments is poorer than 0° [93]. It has been shown that type of fracture of specimens prepared in different raster angles is completely different. In fact, there is a partial failure for the sample printed in a raster angle of 0°, whereas 90°and alternating 0°/90° specimens show a sudden clean fracture [94].

Printing configuration and bead orientation may also have a significant effect on the surface quality of the printed PEEK. According to Wang et al. [84], the horizontal direction provides better surface quality under the same printing conditions. This was attributed to the squeezing action of the nozzle. The Poisson’s ratio can be considered as a criterion for comparing surface quality. Results have shown that printing of PEEK in the vertical direction leads to a lower Poisson’s ratio. Since applied force is normal to the printed layers, the number of voids increases during loading and as a result, this parameter declines [93].

Raster angle can change the roughness and colour of PEEK samples. 3D printing at a horizontal direction and a raster angle of 90° provides a rougher and a whiter surface than 0°. Since the deposition path is shorter, the thermal gradient between beads is lower, and thereby diffusion is faster leading to a rougher surface finish for the part printed at a raster angle of 90° [93].

### 3.2. Printing Layer Thickness

Wang et al. [84] examined the effect of nozzle diameter and printing layer thickness on physical and mechanical properties of PEEK. Results from samples printed with a 0.4 mm nozzle diameter exhibited little change in the density and tensile strength of PEEK. When nozzles with larger diameter were used, the tensile strength deteriorated with an increase in layer thickness. The authors hypothesised that the interlayer bonding strength was weakened for the layers thicker than 0.35 mm. Wu et al. [92] reported that the highest tensile, flexural, and compressive strength could be obtained for PEEK parts at an optimal layer thickness of 0.3 mm. It is noteworthy that they used a custom-built 3D printer with a nozzle diameter of 0.4 mm.

**Figure 8:**
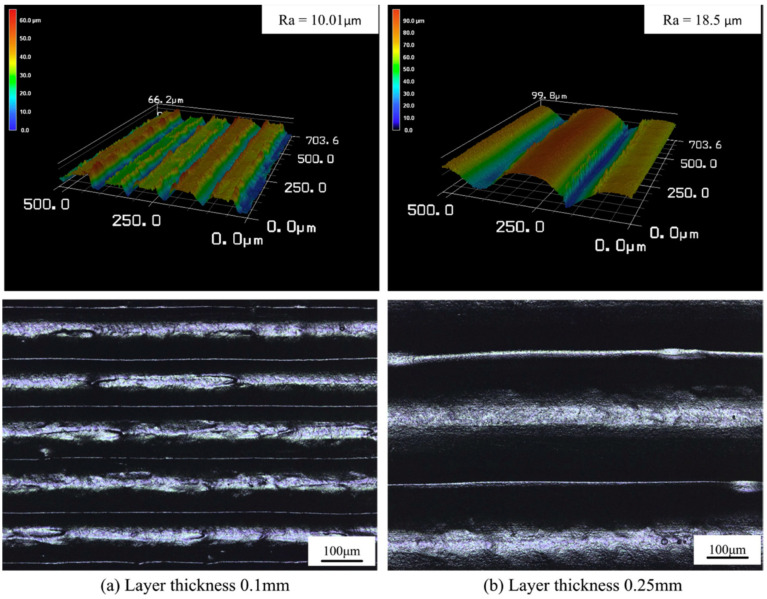
Surface morphology of PEEK samples printed in the vertical direction: layer thickness of (**a**) 0.1 mm, and (**b**) 0.25 mm [84].

Surface roughness (R_z_) shows an increase as layer thickness increases (Figure 8) due to gaps between layers and the enlargement of voids. If the printing layer is thicker than half of nozzle diameter, inappropriate quality can be observed in a vertical direction. Also, there is an apparent step effect when thicker layers are used for printing [84]. Wang et al. [109] predicted surface roughness of PEEK by developing a model. They found that there was a good agreement between the experimental and the predicted surface roughness and finally recommended a layer thickness of 0.15 mm for 3D printing of PEEK.

### 3.3. Processing Conditions

#### 3.3.1. Ambient/Printing Chamber Temperature

**Figure 9:**
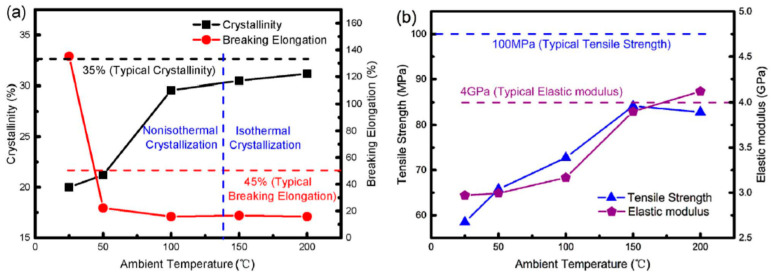
Ambient temperature dependency of (**a**) crystallinity and (**b**) mechanical properties of PEEK samples [88].

In 3D printing, a lower accuracy can be seen for PEEK especially in more complex geometric areas such as the narrow area of tensile specimens owing to some reasons including its high T_g_ and T_m_, high viscosity, shrinkage, etc. The temperature inside of printing chamber can be a determining factor in the production of parts with desirable accuracy and performance. Yang et al. [88] used a temperature-control 3D printing system to study the relationship between ambient/printing chamber temperature in FFF and the performance of PEEK. They found that control of ambient temperature plays a crucial role in the mechanical properties of printed PEEK. As shown in Figure 9, the percentage of crystalline phase increases as ambient temperature increases which in turn leads to a remarkable improvement in Young’s modulus and tensile strength. At a temperature of 200 °C, they obtained a Young’s modulus even higher than that of injection-moulded PEEK.

When the ambient temperature is low, there is a large temperature difference between nozzle and the printing chamber. This provides a rapid cooling for PEEK chains (imperfect crystallisation) and causes significant warping distortion. There is a reverse relationship between ambient temperature and warping distortion in additive manufacturing. Higher temperatures can help to slow down the crystallisation phenomenon and reduce internal stresses [102]. In fact, at high ambient temperatures, polymer chains have adequate time and energy to crystallise, so PEEK can experience a more perfect crystallisation process and thereby higher degree of crystallinity is obtained. These findings demonstrate that one can adjust the crystallinity and stiffness/toughness of PEEK by choosing an optimal ambient temperature.

**Figure 10:**
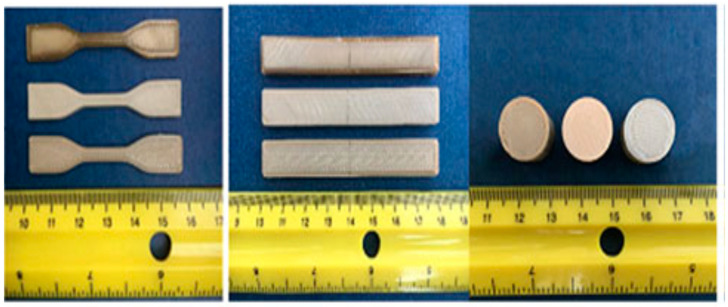
Colour differences in PEEK samples (Hyrel, Intamsys, and Indmatec from top to bottom or from left to right, respectively) [90].

In a study by Wang et al. [90], the effect of ambient temperature on the physical and mechanical properties of PEEK was investigated through printing using three different printers namely, Hyrel Hydra 16AS (Hyrel 3D Ltd., Norcross, USA), Indmatec HPP 155 (Apium Additive Technologies GmbH, Karlsruhe, Germany) and Intamsys FUNMAT HT (Intamsys Technology Ltd., Shanghai, China). The difference among printed samples was visibly evident based on their colour, as illustrated in Figure 10. Additionally, X-ray diffraction (XRD) patterns revealed that the sample printed using the Hyrel, had a completely different microstructure. It showed the lowest degree of crystallinity owing to the fact that the ambient temperature in this printer was 23 °C. In contrast, an ambient temperature of 145 °C in Intamsys printer enabled the PEEK chains to complete the crystallisation process. Even though the ambient temperature was 23 °C in the case of Indmatec, but presence of an adaptive heating system provided a normal crystallisation. These variations in crystallinity affected the mechanical and dynamic-mechanical properties of PEEK. Furthermore, a change (around 10 °C) in glass transition temperature (T_g_) indicates that non-crystalline regions of PEEK can also be influenced by process conditions such as ambient temperature.

To improve the printing conditions, Hu et al. [108] recently developed a modified FFF printer suitable for additive manufacturing of PEEK by adding a heat collector (HC) and a high-power heater to the nozzle module and designing a near fully-enclosed chamber. They found that overall temperature around the nozzle increases by adding this heat collector. A comparison of temperature profiles around the nozzle shows that within 50 mm of the nozzle, there was a large temperature difference (around 50 °C) between improved and traditional nozzles. This was beneficial for 3D printing of PEEK, because it provides a more uniform temperature distribution. According to the authors, the sample printed using traditional nozzle shows severe warpage (20.4%). An increase in ambient temperature can significantly solve this problem and reduce the warpage rate to 6.1%, but there was still severe delamination. By employing the heat collector, the prepared PEEK samples had a warpage rate of 5%, higher crystallinity, and no delamination, which resulted in improved tensile and flexural properties.

#### 3.3.2. Nozzle Temperature

The impact of nozzle temperature on characteristics of printed PEEK is a complicated one, as it can influence the melting of crystalline areas, crystallisation process, and interfacial strength between printing beads directly or indirectly. Moreover, nozzle temperature determines the performance of the printing process for PEEK. If the printing temperature is not high enough, nozzle clogging and delamination of deposited layers can occur. On the other hand, high temperatures may cause thermal degradation of PEEK. Dimensional inaccuracy is another issue which is due to considerable change in viscosity [103] which can change due to fluctuations in the nozzle temperature.

**Figure 11:**
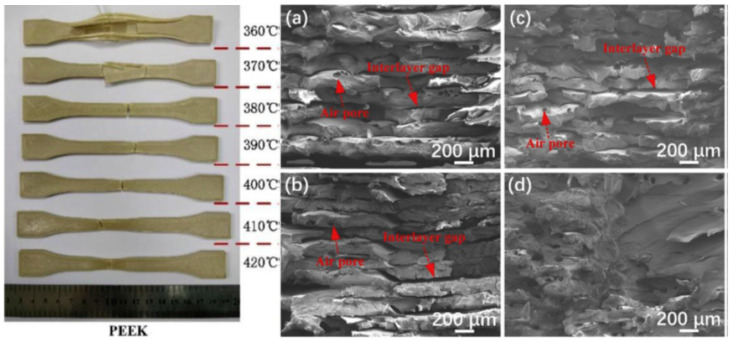
Morphology of the PEEK tensile samples printed at different nozzle temperatures (**left**), and SEM micrographs of tensile fracture surface of printed PEEK at different printing temperatures: (**a**) 370 °C, (**b**) 380 °C, (**c**) 390 °C, and (**d**) 420 °C [96].

The results of some studies [84,88,113,114] show that PEEK has a higher crystallinity and tensile properties when it is printed at higher nozzle temperatures. Table 5 summarises the tensile strength of PEEK at different nozzle temperatures. A high nozzle temperature provides appropriate conditions for chain crystallisation. Since mechanical properties are mostly governed by the crystalline phase in semi-crystalline polymers, this can improve elastic modulus and tensile strength of PEEK. Analysis of fracture surface can give more information about the role of printing temperature on the tensile behaviour of PEEK. Spallation phenomenon occurs for PEEK parts printed at low temperatures (Figure 11). Presence of interlayer gaps could be the main reason for the inferior behaviour of these samples in tensile testing, as illustrated in SEM micrographs (Figure 11). For the samples processed at 420 °C, there is no stratification on the fracture surface, and interlaminar bonding reaches its maximum [96]. It is likely that at these temperatures, the molten filament causes the previous layer to partially melt, thereby increasing the adhesion between layers.

Ding et al. [96] reported a different trend for mechanical properties of PEEK, as shown in Table 5, where a decrease in tensile strength in temperatures between 360–380 °C was reported. Furthermore, the values of tensile strength reported by Ding et al. [96] were higher than those reported by other authors [84,88]. These differences appear to originate from differences in the printing parameters. Comparison between the study conducted by Ding et al. [96] with Yang et al. [88] shows that nozzle diameter and layer thickness are the same, but printing speed and filling direction were different. It can be concluded that lower printing speed (20 mm/s as compared to 40 mm/s) leads to much higher density and tensile strength.

**Figure 12:**
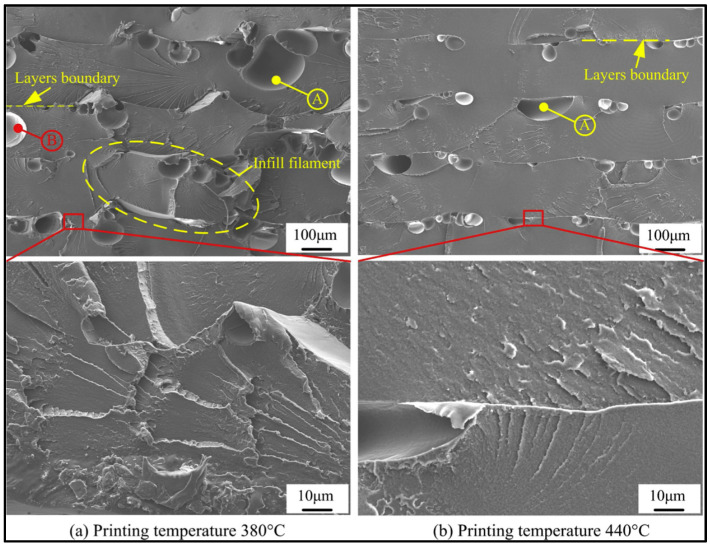
Representative SEM micrographs of the fracture surfaces of PEEK parts printed at different printing temperatures (**a**) at 380 °C (**b**) at 440 °C. A and B indicate voids between layers and a void between the infill filaments [84].

The density of PEEK also increases by applying higher nozzle temperatures. This can be attributed to better fluidity of PEEK, which facilitates filling voids and pores [84]. SEM micrographs (Figure 12) of PEEK samples printed at 380 and 440 °C clearly show the effect of processing temperature on the formation of pores and internal defects. As seen, higher nozzle temperatures result in a reduction in the number and size of the voids.

**Figure 13:**
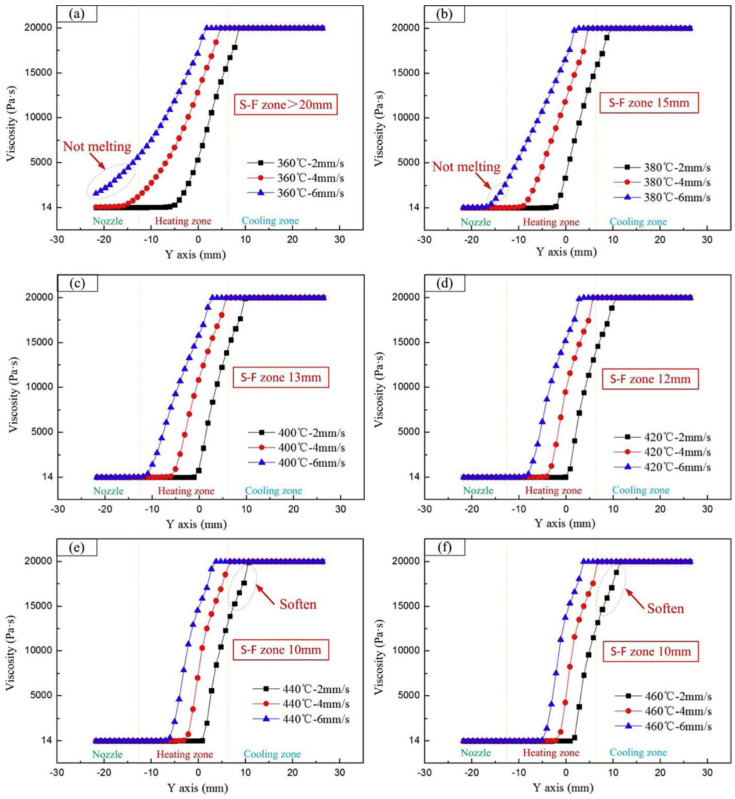
Viscosity of PEEK at various heating temperatures and wire feeding speeds (2, 4 and 6 mm/s) (**a**) at 320 °C (**b**) at 380 °C (**c**) at 400 °C (**d**) at 420 °C (**e**) at 440 °C and (**f**) at 460 °C [84].

It should be noted that there is a limitation for increasing processing temperature due to possible thermal degradation of PEEK. This way, a nozzle temperature in the range of 420–440 °C is recommended for FFF 3D printing of PEEK. On the other hand, fluidity and melting behaviour of the polymer in the flow channel is influenced by the temperature of the printing head. Therefore, the selection of appropriate nozzle temperature is of great importance in an ideal 3D printing process for PEEK. Figure 13 illustrates the viscosity of PEEK at different temperatures and filament feeding speeds. A low nozzle temperature (360 °C) doesn’t provide enough heat to melt PEEK inside the flow channel and thereby, a length of solid-fluid (S-F) zone of >20 mm was observed. Applying a higher temperature (>360 °C) decreases the length of this zone, and it reaches to around 10 mm. Furthermore, the feeding speed of PEEK filament should be optimised to prevent softening of polymer in the cooling zone of the flow channel. Based on these experiments, the authors [84] suggested that a printing temperature of 380–440 °C and a feeding speed of 4 mm/s are the best levels for FFF 3D printing of PEEK.

To address issues relating to 3D printing of highly viscous polymers such as PEEK, with a view of reaching near bulk mechanical properties, a screw extrusion-based additive manufacturing system has been developed and reported in the literature [86]. The authors achieved a Young’s modulus, and tensile strength of 3.93 GPa and 94 MPa for PEEK printed at 390 °C, respectively. The ratio of these values to those of bulk PEEK is 98% and 96%, respectively. Besides, the porosity of the PEEK sample is 2.6% which was much lower than the values reported in other works [103]. These results can be attributed to the role of the extrusion process of this new printing machine that can apply a shear strain on the PEEK chains, leading to high crystallinity. The degree of crystallinity of PEEK samples in this work (39–40%) was nearly doubled compared to injection moulded PEEK (21%).

PEEK showed the highest impact strength at an optimal temperature of 390 °C [96]. Although interlaminar bonding was stronger at higher temperatures, it appeared that a specific layered structure created in the printing of PEEK at 390 °C could release impact energy more efficiently. Moreover, the formation of a more perfect crystalline phase at higher printing temperatures has a detrimental effect on the toughness of PEEK.

Higher nozzle temperatures lead to a lower surface roughness for PEEK parts printed in both horizontal and vertical directions [84]. This may be due to better diffusion of PEEK macromolecules and surface infiltration at higher temperatures.

#### 3.3.3. Bed Temperature

One of the printing parameters that controls the quality of 3D printed parts is bed or substrate temperature. Unfortunately, there is no report in the literature studying the role of this parameter in the performance of PEEK parts using various physio-mechanical tests. However, to determine suitable bed temperature in the FFF process of each polymer, Tseng et al. [86] measured the coefficient of adhesion friction (μs). There was an optimal bed temperature for PEEK at which μs showed the highest value. Higher temperatures weaken the adhesion process due to incomplete solidification of the polymer layer. Based on these criteria, Tseng et al. [86] chose 280 °C for bed temperature in the printing of PEEK samples.

**Figure 14:**
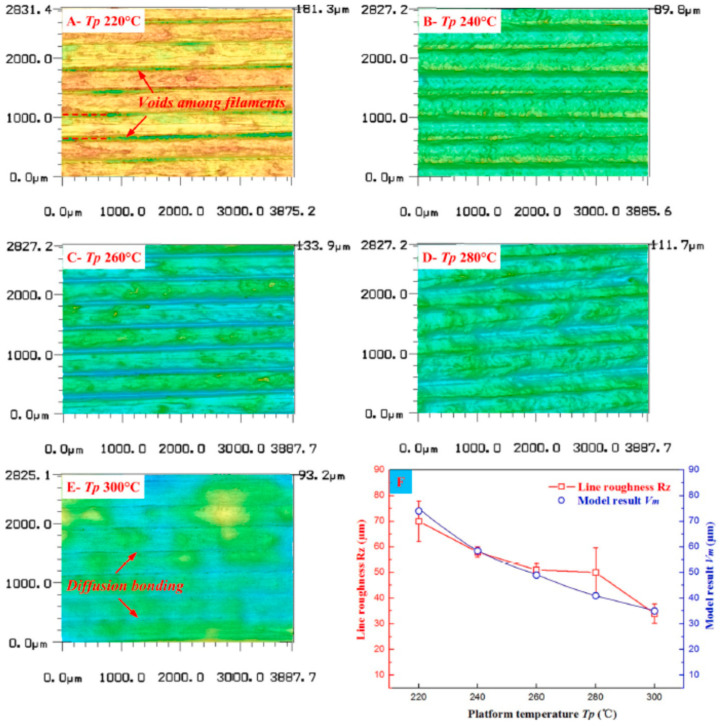
Surface topography of the printed PEEK parts at bed temperatures of (**A**) 220 °C, (**B**) 240 °C, (**C**) 260 °C, (**D**) 280 °C (**E**) 300 °C and (**F**) experimental results and model results of surface roughness in different platform temperatures [109].

In terms of surface quality, Wang et al. [109] developed a model for prediction of surface roughness of PEEK parts prepared via FFF-3D printing. They hypothesised that relationship between molecular diffusion, cross-sectional shape, and heat transfer constitutes a closed-loop. Initially, molecular diffusion and neck growth is fast, but when the temperature drops down, this process slows down, and it reaches its maximum at 230 °C for PEEK. They also studied the effect of some printing parameters on the surface roughness of the PEEK measured by laser scanning microscopy. The experimental results were in a good agreement with the predictions of the model. Figure 14 depicts the effect of bed temperature on the surface topography of PEEK parts. The samples printed at higher temperatures showed smoother morphology and better interfacial bonding among adjacent filaments. This originated from the fact that molecular diffusion continues for a longer time at higher bed temperatures leading to a decrease in the number and volume of voids [109]. However, comparison between bed temperature and nozzle temperature reveals that the former has a less significant effect on the surface roughness.

#### 3.3.4. Printing Speed

**Figure 15:**
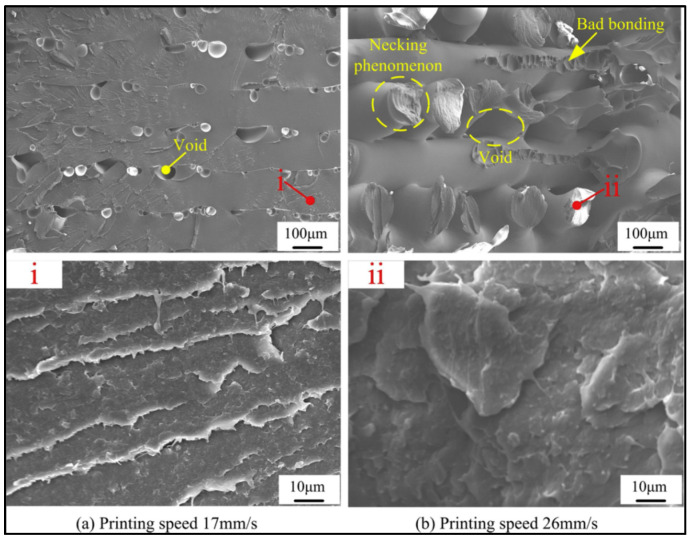
SEM micrographs of tensile-fracture surfaces of PEEK samples printed under different speeds (**a**) at 17 mm/s and (**b**) at 26 mm/s [84].

Generally, there is a reverse relationship between printing speed and mechanical properties of printed parts. In a study by Wang et al. [84], a printing speed of 20 mm/s led to the best tensile strength and increasing this parameter caused a loss in mechanical properties of PEEK. This has been ascribed to the fact that higher speeds don’t provide sufficient time for layers and infill filaments to diffuse and crystallise. The effect of print speed on the fracture surface of the PEEK samples can be seen in Figure 15. The formation of a large number of big voids and weak bonding are the characteristics of the sample printed at higher speeds.

Basgul and colleagues [91] printed a standardised PEEK lumbar fusion cage and found that printing speeds lower than 25 mm/s should be employed. The highest compressive properties were achieved when lower speeds were chosen for 3D printing of PEEK. Porosity in printed items is created by the expansion of voids and entrapping air bubbles in filaments [103]. According to the authors, printing speed had a negative effect on the porosity of the part. No change in crystallinity was observed by increasing printing speed. As a result, they concluded that imperfect filament-to-filament bonding formed at high speeds which was the main reason for the deterioration of the mechanical properties of PEEK.

**Figure 16:**
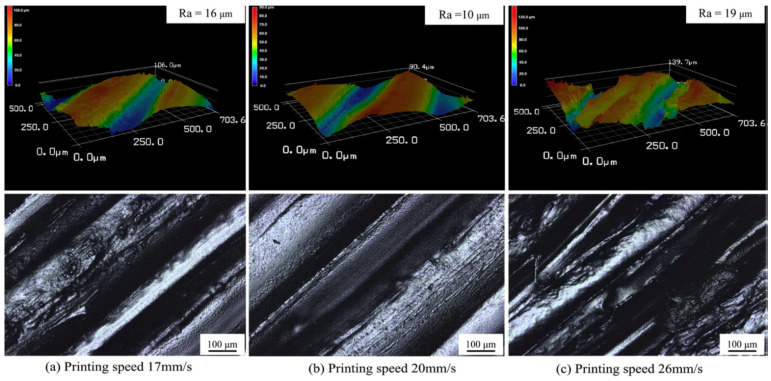
Surface topography of the PEEK parts printed under different printing speeds (**a**) at 17 mm/s (**b**) at 20 mm/s and (**c**) at 26 mm/s [84].

In terms of surface quality, an optimal speed should be used for 3D printing of PEEK. Wang et al. [109] reported that a printing speed of 15 mm/s was appropriate for PEEK. The surface topography demonstrates a large difference among samples printed at different speeds (Figure 16). At low printing speeds, signs of stacking on the surface can be observed. An interaction between the nozzle and the molten filaments (nozzle squeezing) occurs at higher speeds which adversely effects the surface quality. In another study [84], the same authors found that nozzle diameter can influence the effect of print speed on the quality of printed PEEK. When a nozzle diameter of 0.4 mm was used, there was an optimal speed to reach the minimum roughness, while for nozzles with larger diameters, surface roughness slightly increased with printing speed. For nozzles with small diameters, thinner printing layers were used, which were more sensitive to changes in nozzle speeds.

Geng et al. [95] addressed the effects of the extrusion speed and printing speed on the dimensions of an extruded PEEK filament in 3D printing. According to the results, high extrusion speed causes severe fluctuations in the diameter of extruded filaments. It indicates the key role of this factor in controlling dimensional accuracy and surface morphology. There is a similar observation in work by Zhao et al. [69]. They designed orthogonal experiments to optimise the mechanical strength of PEEK for medical applications. Their experiments confirmed that the deviation in diameter of PEEK filament plays an important role in 3D printing.

#### 3.3.5. Heat Treatment Methods

Various heat treatment methods can cause a quite remarkable difference in the crystallisation of FFF printed PEEK parts. Findings of a study by Yang et al. [88] who compared the role of furnace cooling, annealing, air cooling, quenching, and tempering methods reveals that a more perfect crystallisation process occurred using the first two methods. Conversely, air cooling and quenching couldn’t provide an appropriate condition for normal crystallisation, thus led to the lowest crystallinity. The results of mechanical testing indicated that annealing and tempering methods were the most efficient post-processing techniques. It seems that heat treatment methods not only control the crystallisation of PEEK, but also determine the level of residual stress in the printed items. Basgul et al. [111] printed lumbar spinal cages via FFF at different speeds (1500 and 2000 mm/min) and then annealed them at 200 °C or 300 °C. They observed a 14% increase in compression strength of the sample printed at the slower speed. Moreover, it was found that although annealing changes the structure of the pores, it did not reduce the porosity formed during printing.

Recently, 3D printer manufacturers have tried to find new solutions for customising degree of crystallinity. 3DGence has adopted a new approach (rapid cooling/annealing) to control crystallinity of PEEK. An amorphous PEEK part is 3D-printed in the first step, which has a less shrinkage and better distribution of stress. Then it is subjected to annealing to obtain a semi-crystalline final part [115]. Stratasys have been granted a US patent [116] for a 3D printing method that inhibits PEEK crystallisation. In this disclosure, the 3D part material includes a miscible blend of one or more semi-crystalline polymers and one or more secondary materials. Polyetherimides (PEI)/semi–crystalline PEEK (60–80 wt.%) and amorphous polyaryletherketones (50–70%)/semi–crystalline polyaryletherketones blends are among the claimed formulations. It is believed that miscibility allows the amorphous phase to impede the semi-crystalline polymer from forming crystalline regions. The additive manufacturing is followed by a post-processing step involving annealing at a temperature between glass transition temperature and cold crystallisation temperature of the part material. Unlike layer-by-layer crystallisation during 3D printing, which causes curling effects, post-printing crystallisation leads to a uniform shrinkage similar to injection moulding process. Furthermore, annealing provides an opportunity for 3D printed part to be further crystallised without any restriction by non-shrinkable building plate.

## 4. Thermal and Irradiation Degradation

The average peak heat release rate (PHRR) of PEEK is 303 W·g^−1^ which is eight times lower than that of polyethylene (PE). Furthermore, the onset heat release rate temperature (flash point), PHRR, and limiting oxygen index (LOI) of PEEK are 601, 619, and 37.3%, respectively [117]. Having such as superior heat stability allows PEEK to be used in specific applications and has great potential to replace metallic parts on the ISS.

**Figure 17:**
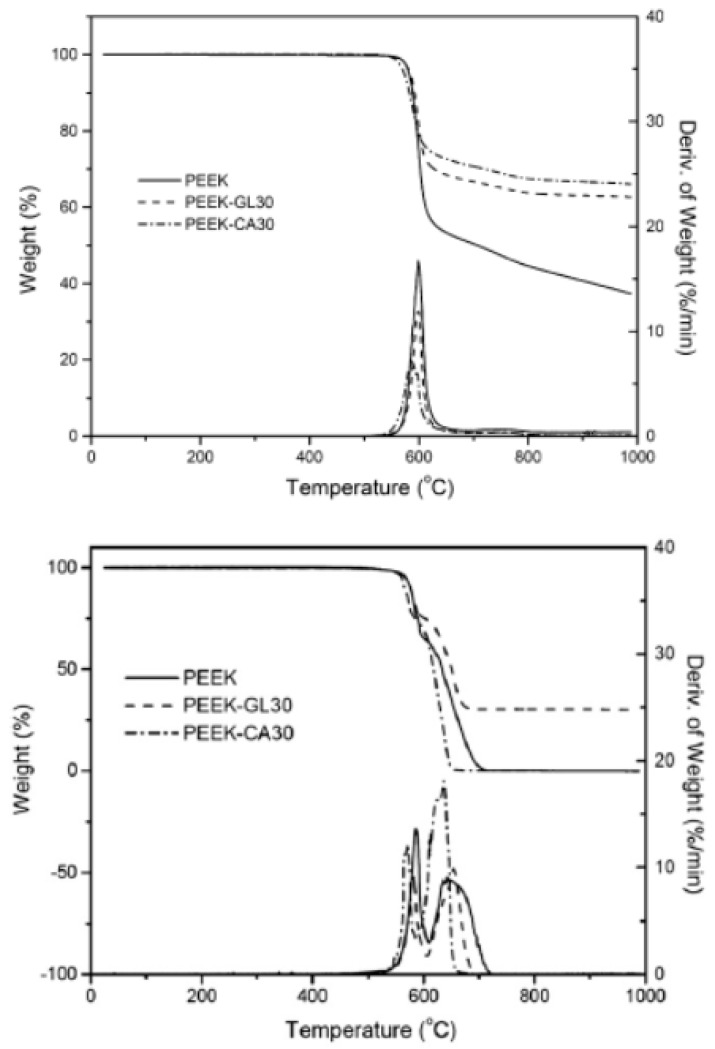
Thermal decomposition of PEEK and its glass (GL30) and carbon (CA30) fibre composites under nitrogen (**top**) and in air (**bottom**) [119].

**Figure 18:**
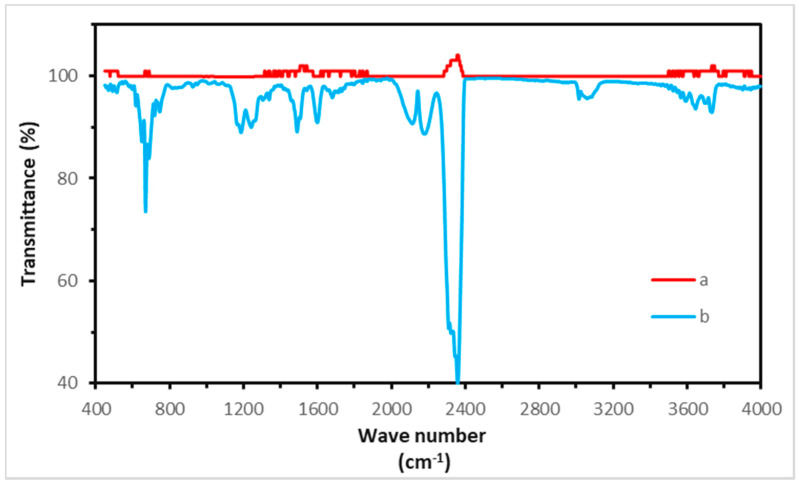
Fourier Transform Infrared spectra of PEEK: (**a**) before start of degradation, and (**b**) at maximum rate of weight loss.

PEEK exhibits a two-step weight loss in an inert environment (Figure 17). The first stage occurs at a temperature range of 500–600 °C and it is attributed to hemolytic polymer chain scission which leads to formation of phenols, carbon monoxide, and carbon dioxide. The former if produced can cause chemical burns which is a concern for space flight applications. In the second step (above 600 °C), polymer residue undergoes cracking and dehydrogenation. Figure 18 shows the FT-IR spectra of PEEK before the start of degradation and at maximum rate of weight loss. The mass loss during first and second steps of thermal degradation of PEEK in nitrogen environment, and char yield at 1000 °C are around 40–45%, 10%, and 45–50%, respectively. Thermal decomposition of PEEK in oxidative atmosphere includes two fast, consecutive stages. Cleavage of ether and ketone bonds begins at temperatures below 500 °C. Ether bonds undergo degradation at lower temperatures, while decomposition of carbonyl bonds mostly occurs at higher temperatures. Thermal oxidation of carbonaceous material takes place in the next step, leaving no char at 650–700 °C [118,119,120].

A summary of the thermal degradation parameters for PEEK (obtained from TGA testing) are presented in Table 6. T_onset_ is onset degradation temperature. T_10%_ and T_max_ are the temperatures corresponding to 10% weight loss and maximum degradation rate, respectively. According to the data, oxidative degradation starts at 460–480 °C which is 40–50 °C lower than that of degradation in an inert atmosphere. Furthermore, Tsai et al. [121] studied thermal decomposition of PEEK using flash pyrolysis gas chromatography/mass spectrometry (pyGC/MS) and found that 1,4-diphenoxybenzene and 4-phenoxyphenol are the decomposition products at 450 °C. This indicates that 3D printing of PEEK in the space environment should be performed at temperature lower than this. Based on these findings, it is reasonable to set a maximum temperature of 440 °C to not only achieve an efficient printing process, but also avoid any probable thermal degradation.

Vaezi et al. [103] tested different extrusion temperatures (350–450 °C) and observed colour change or entrapped voids inside PEEK samples. According to the results, a temperature range of 400–430 °C was suggested by the authors for additive manufacturing of PEEK. To address the possibility of degradation of PEEK during FFF 3D printing, Ding et al. [96] printed PEEK at 420 °C and then compared the FT-IR spectra of PEEK filament and the final part. The printed sample showed the PEEK’s characteristic absorption peaks (C = O at 1651 cm^−1^, -O- at 1186 cm^−1^, and C-O at 1224 cm^−1^) with same intensity, so they concluded that there was no thermal decomposition in printing of PEEK at this temperature. The same results were reported by Zhao [69] where the authors comparing FT-IR spectra of the PEEK granule, filament, and samples printed at 375 °C for medical applications.

Aiming to use PEEK in an out-off-earth environment and on ISS, irradiation effect on this high-performance polymer must be considered. It has been shown by Mylläri et al. [125] that UV irradiation for 1056 h had a little effect on strength and elastic modulus of PEEK fibres, while it significantly deteriorated elongation at break due to random scission of the chains. There are also other works [126,127] that reported even an increase in yield strength of PEEK sheets caused by crosslinking. Although an increase in T_g_ and zero shear viscosity of PEEK could be seen by increasing irradiation time, but crystallinity and melting temperature are not influenced by this parameter [125].

Nakamura et al. [128] examined the effect of low Earth orbit (*LEO*) environment on the PEEK films and concluded that UV constituent in LEO is the main reason for surface browning and crosslinking phenomenon. They also observed that atomic oxygen (AO) in ISS orbit may cause surface erosion and a decrease in thickness. An interesting finding was that the degree of degradation was much smaller than the predicted values. The authors attributed it to the attitude change of the ISS during flight and formation of SiO_x_ layer on surface of the samples. This layer has a low light transmission and can prevent detrimental effect of AO.

These results show that stabilisation is a critical part of development of PEEK-based devices. This way, it seems incorporating appropriate anti-aging additives into PEEK bulk is as one of the best solutions.

## 5. Conclusions

This review has examined FFF 3D-printing of PEEK with an aim to serve as a foundation for the development of 3D printed PEEK parts in the international Space Station. Here, the focus was on understanding process-structure-property relationships in printing of this high-performance plastic. The effects of various printing parameters including building orientation, nozzle temperature, ambient temperature, bed temperature, layer thickness, print speed, and heat treatment on the physical/mechanical properties and print quality were clarified. Based on the findings of published works, the recommended levels of the parameters in order to approximate the performance of conventionally moulded samples are presented in Table 7. However, it is important to note that these are based on tensile mechanical performance mostly.

The review of the reported literature revealed some promising results for mechanical properties of PEEK samples printed via FFF process. However, there are still many challenges regarding 3D printing of PEEK, which needs to be taken into account. From technical point of view, optimising the temperature difference between nozzle and bed/chamber in 3D-printers is the most critical aspect. In fact, thermal gradients across beads should be minimised to fabricate parts with desirable mechanical performance and dimensional stability. Therefore, development of a customised printer considering this point is necessary for 3D printing of PEEK. On the other hand, it appears that there is a strong need for more experimental and modelling research on the microstructure, fracture phenomenon, rheological behaviour, and crystallisation of PEEK. Moreover, the interactive effects of the printing parameters should also be studied by the wider research community.

## Figures and Tables

**Table 1:** Physical and mechanical properties of PEEK [77].

Material Properties	Typical Value
Density	1.3 g/cm^3^
Melting temperature	343 °C
Glass transition temperature	143 °C
Coefficient of thermal expansion	Average below Tg: 55 ppm/k Average above Tg: 140 ppm/k
Heat deflection temperature	152 °C
Thermal conductivity	0.32 W/ m.k
Young’s modulus	4 GPa
Tensile strength	100 MPa
Elongation at break	45%
Flexural modulus	3.9 GPa
Flexural strength	162 MPa
Compressive modulus	3.2 GPa
Compressive strength	125 MPa
Shore D hardness	84.5
Water absorption	0.45%
Flammability	V-0

**Table 2:** 3D printing parameters of PEEK via fused filament fabrication (FFF).

Study	PEEK	Printing Machine	Nozzle Temperature (°C)	Nozzle Diameter (mm)	Printing Speed (mm/s)	Bed Temperature (°C)	Layer Thickness (mm)	Infill Density (%)
Basgul, 2018 [91]	PEEK OPTIMA™ LT1	Indmatec HPP 155/Gen 2	390–410	0.4	1000–3000 mm/min	100	1st layer height: 0.1 Top solid layers: 3	100
Wu, 2015 [92]	PEEK Jilin University	custom-build printer	-	0.4	-	-	0.2–0.4	-
Yang, 2017 [88]	VictrexPEEK 450G	custom-build printer	360–480	0.4	40	-	0.2	-
Arif, 2018 [93]	Victrex PEEK 450G	Indmatec HPP 155	410 °C; 1st layer: 390 °C	0.4	800 mm/min; 1st layer: 300 mm/min	100	0.1; 1st layer: 0.18	100
Rahman, 2015 [94]	Arevo Labs	Arevo Labs	340	1.8	50	230	0.25	100
Rinaldi, 2018 [85]	Victrex PEEK 450 PF	Indmatec	400	0.4	20	100	-	20–100
Geng, 2019 [95]	VICTREX 450 G	self-made printing system	360	0.4–0.6	extrusion speed: 0.1–120 mm/min	110	-	-
Ding, 2019 [96]	450G, Junhua, China	custom-build printer	360–420	0.4	20	270	0.2	-
Wang, 2019 [84]	-	custom-build printer	360–460	0.4–0.8	17–26	280	0.1–0.5	-
Deng, 2018 [97]	PEEK-ZhongshanYousheng	custom-build printer	350–370	-	20–60	-	0.2–0.3	20–60
Zhao, 2018 [69]	Victrex PEEK450G	homemade printer	355–375	0.4	30	230–270	0.2	100
Tseng, 2018 [86]	Victrex (PEEK 90G and PEEK 450G)	a new screw extrusion-based 3D printing system	370–390	-	-	280	-	-
Honigmann, 2018 [98]	Apium PEEK 450	Apium P220	Up to 520	0.4	-	Up to 160	0.1–0.3	-
Stepashkin, 2018 [99]	CF-composite Victrex 150 CA30	custom-build printer	380	0.35	100 mm/min	95	0.25	100
Berretta, 2017 [100]	VICTREX^®^ 450G/Plasticyl PEEK 101	MendleMax v2.0	350–390	-	30	300	-	-
Xiaoyon, 2017 [101]	-	-	-	0.4	20	25–130	0.8	100
Wu, 2014 [102]	PEEK Jilin University	custom-build printer	340–360	-	-	150	0.3	-
Daurskikh, 2018 [89]	Victrex PEEK 450G	-	-	-	-	-	-	100
Vaezi, 2015 [103]	Victrex PEEK 450G	-	350–450	-	-	Up to 130	0.2	-
Wang, 2019 [90]	Apium PEEK 450 Natural	Hyrel Hydra 16AS, Indmatec HPP 155, Intamsys FUNMAT HT	400	0.4	20	100	0.1–0.2	100
Cicala, 2017 [104]	Luvocomm	Roboze one 400+	420	-	20	110	0.1	75
Han, 2019 [87]	Victrex PEEK 450G	Jugao-AM Tech. Corp	420	0.4	40	-	0.2	-
Han, 2019 [105]	Evonik VESTAKEEP^®^i4 G	Apium P220	480	0.4	-	130	0.2	-
Gonçalve, 2018 [106]	Victrex PEEK 450G	Indmatec HPP 155	400	0.4	20	100	0.1	100
Li, 2019 [107]	ZYPEEK 550 G	FUNMAT HT	400	0.4	15	160	0.1	100
Hu, 2019 [108]	Sting3d Technology	Modified Speedy Maker	385	0.4	25	135	0.1	100
Wang, 2019 [109]	PEEK 450G, Junhua	-	380–420	0.4	5–25	220–300	0.1–0.3	100
Sviridov, 2019 [110]	Victrex PEEK 381G	TotalZAnyForm 950 PRO HOT+	450	0.7	40	-	0.75	100
Basgul, 2019 [111]	PEEK OPTIMA™ LT1	Indmatec HPP 155/Gen 2	390–410	0.4	1500/2000 mm/min	100	0.1	100
Lafont, 2017 [106] van Egmond [112]	Victrex 450G	Indmatec HPP 155	390	0.4	20	100	0.1	100

**Table 3:** Parameters of 3D printing of PEEK recommended by filament manufacturers.

Brand Name	Nozzle Temperature (°C)	Bed Temperature (°C)	Printing Speed (mm/s)	Chamber Temperature (°C)	Bed Preparation
ThermaX™	375–410	130–145	10–50 for 0.2 mm thickness	70–140	Ultem™ Tape
3D4MAKERS	360–400	120	15–30	70–150	PEI sheet
Polyfluor	335–390	120	-	-	-
KetaSpire^®^	390–420	>200	-	-	-
LUVOCOM	370–420	>120	-	-	-

**Table 4:** Effect of raster layer orientation on the mechanical properties of PEEK.

Building Orientation/Raster Angle	Young’s Modulus (GPa)	Tensile Strength (MPa)	FlexuralStrength (MPa)
Horizontal/0° [93]	3.80	82.58	142.0
Horizontal/90° [93]	3.54	72.88	124.3
Vertical/90° [93]	3.03	9.99	16.40
Horizontal/+45°/−45° [85]	3.98	98.9	-
Vertical/+45°/−45° [85]	1.6	19.6	-
Horizontal/0° [94]	2.83	73.19	114.16
Horizontal/90° [94]	2.69	53.91	78.63
Horizontal/0°/90° [94]	2.73	67.75	95.22
Horizontal/0° [110]	3.29	89.4	-
Horizontal/+45°/−45° [110]	3.03	81.7	-
Horizontal/0°/90° [112]	3.38	78.1	-
Vertical/+45°/−45°	2.21	31.1	-

**Table 5:** Tensile strength of PEEK at different nozzle temperatures.

	Tensile Strength (MPa)						
Study	360 °C	380 °C	400 °C	420 °C	440 °C	460 °C	480 °C
Yang et al. [88]	48.5	49.5	54	59	55	57	55
Wang et al. [84]	-	59	67.5	70	72.5	-	-
Ding et al. [96]	82	79	83	84	-	-	-

**Table 6:** Summary of the thermal degradation parameters (obtained from TGA test) for PEEK.

Study	T_onset_ (°C)	T_10%_ (°C)	T_Max_ (°C)	Heating Rate (°C/min)
[122]	inert: 514 oxidative: 466	inert: 541 oxidative: 512	inert: 554 oxidative: T_max-I_: 517 T_max-II_: 575	10
[118]	inert: 521	inert: 544	inert: 558	10
[123]	inert: 520 oxidative: 478	inert: 544 oxidative: 520	inert: 558 oxidative: T_max-I_: 530 T_max-II_: 584	10
[124]	-	inert: 588 (MFI: 15 g/10 min) 592 (MFI: 27 g/10 min) 599 (MFI: 85 g/10 min)	inert: 590–600	10

**Table 7:** Recommended values of the parameters for FFF 3D-printing of PEEK.

Parameter	Value/Type
Raster angle	0° [93]
Layer thickness	0.1–0.3 mm [92]
Ambient temperature	150–200 °C [88]
Nozzle temperature	420–440 °C [84]
Print speed	20 mm/s [91]
Infill	100%
Heat treatment method	Annealing [88]
